# Combining stable isotope analysis with DNA metabarcoding improves inferences of trophic ecology

**DOI:** 10.1371/journal.pone.0219070

**Published:** 2019-07-22

**Authors:** Melissa R. L. Whitaker, Christopher C. M. Baker, Shayla M. Salzman, Dino J. Martins, Naomi E. Pierce

**Affiliations:** 1 Museum of Comparative Zoology, Department of Organismic and Evolutionary Biology, Harvard University, Cambridge, MA, United States of America; 2 ETHZ Entomological Collection, Department of Environmental Systems Science, ETH Zürich, Zürich, Switzerland; 3 Mpala Research Centre, Nanyuki, Kenya; 4 Department of Ecology and Evolution, Princeton University, Princeton, NJ, United States of America; Universidade de São paulo, BRAZIL

## Abstract

Knowing what animals eat is fundamental to our ability to understand and manage biodiversity and ecosystems, but researchers often must rely on indirect methods to infer trophic position and food intake. Using an approach that combines evidence from stable isotope analysis and DNA metabarcoding, we assessed the diet and trophic position of *Anthene usamba* butterflies, for which there are no known direct observations of larval feeding. An earlier study that analyzed adults rather than caterpillars of *A. usamba* inferred that this butterfly was aphytophagous, but we found that the larval guts of *A. usamba* and two known herbivorous lycaenid species contain chloroplast 16S sequences. Moreover, chloroplast barcoding revealed high sequence similarity between chloroplasts found in *A. usamba* guts and the chloroplasts of the *Vachellia drepanolobium* trees on which the caterpillars live. Stable isotope analysis provided further evidence that *A. usamba* caterpillars feed on *V. drepanolobium*, and the possibilities of strict herbivory versus limited omnivory in this species are discussed. These results highlight the importance of combining multiple approaches and considering ontogeny when using stable isotopes to infer trophic ecology where direct observations are difficult or impossible.

## Introduction

Few aspects of an organism’s ecology are more fundamental than its diet. Trophic interactions directly and indirectly influence species distributions and richness, community composition, population dynamics, ecosystem function, primary productivity, and nutrient cycling [1–3]. Despite the importance of trophic interactions for understanding fundamental aspects of biodiversity and ecosystems, basic dietary data for many animals are often incomplete, erroneous, or missing. Assessing an animal’s diet is a non-trivial task: diets can be highly variable between individuals and populations of the same species, they may fluctuate or vary according to seasons and geography, and they are often highly dependent on other community and ecosystem properties. Direct observation of feeding is often difficult—particularly for animals that are rare, cryptic, dangerous, very small, or found in remote or inaccessible habitats [4, 5]. Even when data based on direct observations are available, such data usually only provide a snapshot of the overall diet.

It is therefore unsurprising that even well-studied organisms are frequently represented by inadequate natural history data. For example, the Lepidoptera (moths and butterflies) are arguably some of the world’s best-known insects, yet significant data gaps remain regarding basic life history traits, particularly larval diets. Missing natural history data are especially problematic for moths, of which many groups remain understudied, but important data gaps exist in butterfly lineages as well, including the family Lycaenidae.

The Lycaenidae is a large family with diverse life histories and diets. Lycaenids are well known for their larval associations with ants, whereby caterpillars produce nutritious secretions from specialized organs to reward and appease ants in exchange for ants’ protective services against predators and parasitoids [6]. Ant-lycaenid interactions are widespread, and although the majority of these associations are considered mutualistic, a number of lycaenid species have switched to explicitly parasitic lifestyles in which caterpillars feed on ant regurgitations (trophallaxis) or directly on ant brood or ant-tended insects. Aphytophagy (feeding on non-plants) has at least 13 independent origins across the lycaenid phylogeny [7], and specialization either by host plant or ant-association can affect population structuring and subsequent evolution [8].

The larval diets of many lycaenid butterflies remain poorly characterized, likely because the caterpillars are camouflaged, difficult to rear in captivity and often live in inaccessible microhabitats such as flower buds or subterranean ant nests. For many species, no records exist for direct observations of feeding behaviors, leaving researchers to rely on indirect evidence to infer larval diets. One such species is *Anthene usamba* (previously *A. hodsoni*), a lycaenid butterfly found in the savannas of eastern Africa.

Female *A. usamba* butterflies oviposit exclusively on *Vachellia drepanolobium* (also called *Acacia drepanolobium*), and preferentially oviposit on plants occupied by *Crematogaster mimosae* ants, a phytoecious species that nests exclusively in *V. drepanolobium* [9]. Colonies of *C. mimosae* occupy swollen stipular thorns (‘domatia’) and are rewarded with extrafloral nectar; in return, the ants defend their host trees from herbivores [10]. *Anthene usamba* caterpillars also reside in the plant domatia, where they complete their development in close association with the ants [11]. Like many ant-associated lycaenids, *A. usamba* caterpillars possess specialized organs to reward and appease ants. Ants actively defend the caterpillars and allow them to live in the domatia, where they are at least partially protected from predators and parasitoids. Each caterpillar chews a small hole in the domatium wall through which the freshly eclosed adult exits before its wings harden.

Because caterpillars of *A. usamba* are found within the plant domatia during the day, they have never been observed feeding. Previous work using stable isotopes to infer the diet of *A. usamba* found that adult butterflies are enriched in ^15^N relative to their host ants, and concluded that *A. usamba* caterpillars are aphytophagous, presumably preying on their ant hosts [9]. However, a later survey of larval gut microbiota across lycaenids [12] showed a surprising abundance of plant material in the guts of *A. usamba* caterpillars, prompting further investigation into their diets. Here, we combine results from DNA metabarcoding of larval gut contents with stable isotope analysis of larval cuticles, with the specific aims of providing a more detailed assessment of larval diet in *A. usamba*, reconciling the seemingly contradictory results of previous examinations of this species’ trophic ecology [9, 12], and and highlighting the advantages of combining multiple indirect approaches to infer diet.

## Materials and methods

### Sampling for gut content analysis

*Anthene usamba* caterpillars that were third instar or older were collected in June 2014 by surveying the domatia of *V. drepanolobium* trees at field sites on Suyian Ranch in Laikipia County, Kenya (0°31.3'N 36°43.3'E, 1880m asl). Caterpillars were collected and placed in a petri dish with no food or ants for a minimum of 5 hours to reduce the amount of digested and partially digested food in their guts. Following the fasting period, most caterpillars were killed in ethanol, rinsed in sterile phosphate buffered saline, and their guts were dissected using flame-sterilized dissecting tools. Dissected guts were preserved in 97% ethanol and the remaining tissues were dried in vials with Drierite indicating desiccant (CaSO_4_ desiccant; W.A. Hammond Drierite Co. Ltd, Xenia OH) for stable isotope analysis (see below). Three *A. usamba* caterpillars were not starved or dissected but were preserved whole; these samples were surfaced sterilized using 10% bleach solution (following [12] & [13]) prior to DNA extraction and were not included in the stable isotope analyses.

For each of the dissected caterpillars, leaf tissue and ant workers were collected from the same tree as the caterpillar, and were dried in desiccant. When present, herbivores (scale insects), ant brood, and spiders were also collected and dried. Since lycaenid caterpillars can be challenging to identify morphologically (e.g. Baker *et al*., 2016 [14]), we verified that these were *A. usamba* using molecular barcoding (S1 File).

To allow for comparisons to lycaenids with known diets, caterpillars of four additional lycaenid species were included in gut content 16S analysis (detailed collection information is given in Table 1). Caterpillars of *Miletus biggsii*, an entomophagous species that feeds on aphids, and *Flos apidanus*, a phytophagous species that feeds on *Lithocarpus* leaves [15], were collected in Singapore in July 2014. Caterpillars of *Aloeides pallida*, which feed on ant brood [16, 17], and *Azanus natalensis* (species determined by barcoding; see supporting information) found feeding on *Vachellia* (species undetermined) leaves, were collected in South Africa in September 2014. Samples were collected under permits NP/RP14-057 (National Parks Board, Singapore) and 0011-AAA008-00064 (CapeNature, South Africa). When possible, caterpillars were starved and dissected (‘gut’ samples in Table 1), but in some cases caterpillars were preserved whole and surface sterilized (‘whole caterpillars’ in Table 1) prior to DNA extraction.

**Table 1:** Samples included in 16S amplicon sequencing. G: guts, WC: whole caterpillars, P: phytophagous, A: aphytophagous.

Species	Tissue Types (n)	Collection Location	Diet
*Anthene usamba*	G (14), WC (3)	Kenya	under investigation
*Azanus natalensis*	WC (2)	South Africa	**P**: *Vachellia* leaves
*Flos apidanus*	G (6)	Singapore	**P**: *Lithocarpus* leaves
*Miletus biggsii*	G (3), WC (1)	Singapore	**A**: aphids
*Aloeides pallida*	G (2)	South Africa	**A**: ant brood

### 16S amplicon sequencing and analysis

DNA was extracted using the PowerSoil DNA Isolation Kit (MoBio Laboratories, Carlsbad CA), according to the manufacturer’s protocol, with the addition of a proteinase-K lysis step prior to cell disruption by bead-beating. Extracted DNA was sent to Argonne National Laboratories (Lemont, IL) for library preparation and sequencing of the V4 region of the 16S rRNA gene. Amplicon libraries were prepared using barcoded primers 515F (5'-GTGYCAGCMGCCGCGGTAA-3') and 806R (5'-GGACTACNVGGGTWTCTAAT-3') according to previously published methods [18]. Libraries were pooled and sequenced on an Illumina MiSeq sequencer using 150 bp paired-end sequencing technology.

The sequence library was demultiplexed using QIIME version 1.8.0 [18] with a minimum Phred score of 20. Due to reduced read quality in reverse reads, only forward reads were used. Sequences were de novo clustered at 97% identity into Operational Taxonomic Units (OTUs) using UPARSE [19], and chimeric sequences were removed using UCHIME [20] and the GOLD reference database [21]. Taxonomy was assigned to clusters within QIIME using the RDP classifier [22] trained on the Greengenes database version 13_8 [23] with default confidence levels. Representative sequences were then aligned using PyNast [24] and a phylogenetic tree was constructed using FastTree [25] implemented in QIIME.

The resulting biom table [26], phylogenetic tree, and mapping file were imported into R [27] as a phyloseq data object using the phyloseq package [28]. Non-bacterial OTUs (e.g., Archaea and unclassified sequences) and OTUs with fewer than 10 sequences overall or comprising less than 0.01% of a sample’s total library were removed, and samples represented by fewer than 500 sequences were excluded from analysis. The biom table, associated metadata, and analysis code are available via the Harvard Dataverse (DOI: 10.7910/DVN/YCZ76H).

Due to ancient homology between bacterial 16S and eukaryotic organellar 16S sequences, it is common for chloroplast and mitochondrial sequences to co-amplify with bacterial sequences when using universal bacterial primers [29, 30]. We compared the prevalence of chloroplast sequences in the sequence libraries of *A. usamba* to those of aphytophagous and phytophagous lycaenid species. Proportions of chloroplast to non-chloroplast sequences were calculated for each sample, and non-parametric Wilcoxon rank sum tests and Kruskal-Wallis tests (corrected for multiple comparisons) were used to compare chloroplast prevalence between tissue types and diets using the *stats* and *pgirmess* packages [27, 31].

### Plant barcoding

We used extracted DNA from the guts of 12 *A. usamba* caterpillars to identify ingested plant material using plant barcoding of the trnL chloroplast gene. We used the primer pairs c & d described by Taberlet *et al*. (2007) [32] to amplify 565 bp with the following PCR protocol: initial denature at 95°C for 3 minutes, 36 cycles of a 1 minute 95°C denature, 1 minute 58°C anneal, and 2 minute 72°C extension followed by a final extension of 72°C for 5 minutes. PCR products were cleaned using the ExoSAP-IT method and sequenced on an ABI 3730xl sequencer using the services provided by Eton Bioscience (Boston, MA, USA). Raw sequences were edited in Geneious version 9.1.3 [33] and identified using BLAST against Genbank [34]. Edited sequences were deposited in Genbank under accession numbers MK263466—MK263477.

### Stable isotope analysis

We conducted carbon and nitrogen stable isotope analysis of 17 *A. usamba* larval samples (14 of which were included in the 16S analysis, plus larval cuticles of three additional specimens). For comparison, we also analyzed samples of *V. drepanolobium* leaf tissue, and of *C. mimosae* ant larvae, pupae and workers (Table 2). To place these in a broader trophic context, we analyzed samples of scale insects found inside *C. mimosae*-occupied domatia as examples of known herbivores, and samples of two species of spiders as examples of known carnivores. All of these samples were collected at Suyian in June, 2014.

**Table 2:** Samples included in stable isotope analysis.

Organism	Description	Number
*Vachellia drepanolobium*	leaflets	47
Unidentified scale insects	known herbivores	15
*Anthene usamba*	larval cuticles	17
*Crematogaster mimosae*	larvae	20
	pupae	20
	workers	30
Unidentified *Clubiona* sp.	spiders (known carnivores)	3
Unidentified Salticidae sp.	spiders (known carnivores)	11

Tissues were stored in desiccant until they could be processed in the lab, with desiccant changed as necessary to ensure that the samples dried completely. For each sample, we weighed approximately 4 mg of plant leaflets or 1 mg of insect or spider tissue into a tinfoil capsule (5x9 mm capsules, Costech Analytical, Valencia CA) in preparation for analysis. *V. drepanolobium* leaflets or *C. mimosae* ants were pooled from the same tree or colony where necessary to achieve the target mass. We avoided sampling from abdomens or guts to avoid any influence of gut contents on the data [35], with the exception of the *C. mimosae* larvae, which were impractical to dissect and were processed whole.

Samples were sent to the UC Davis Stable Isotope Facility for analysis. Each sample was analyzed for carbon and nitrogen isotopes with a PDZ Europa ANCA-GSL elemental analyzer interfaced to a PDZ Europa 20-20 isotope ratio mass spectrometer (Sercon Ltd., Cheshire, UK). Throughout this paper, δ^15^N stable isotope ratios for nitrogen are expressed relative to atmospheric air, and δ^13^C stable isotope ratios for carbon relative to Vienna PeeDee Belemnite.

Since our sequencing data suggested an abundance of host plant DNA, we used our stable isotope data to re-examine the conclusion of previously published work [9] that *A. usamba* caterpillars feed on *C. mimosae* larvae. We used Student *t*-tests to compare δ^15^N and δ^13^C values among caterpillars, *C. mimosae* larvae, and *V. drepanolobium* host plants. Furthermore, we used ANOVAs to test our expectation that *C. mimosae* larvae, pupae and workers would show similar δ^15^N and δ^13^C values. Raw stable isotope values and analysis code are available via the Harvard Dataverse (DOI: https://doi.org/10.7910/DVN/YCZ76H).

As δ^15^N is often used as a trophic level marker [35–38], we compared *Anthene*’s δ^15^N against scale insects, as known herbivores, and spiders, as known carnivores. Such comparisons take advantage of the empirical consistency in isotope fractionation between trophic levels, i.e.

$$\begin{array}{cc} \Delta^{15}{\text{N}}_{\text{consumer}} & \equiv \delta^{15}{\text{N}}_{\text{consumer}}-\delta^{15}{\text{N}}_{\text{diet}} \end{array}$$ (1)

$$\begin{array}{cc} \Delta^{13}{\text{C}}_{\text{consumer}} & \equiv \delta^{13}{\text{C}}_{\text{consumer}}-\delta^{13}{\text{C}}_{\text{diet}}. \end{array}$$ (2)

Nitrogen is generally more useful than carbon for assessing trophic position (e.g. [39]), because typical Δ^15^N values in the order of 3-4‰ provide reasonable separation between trophic levels, while Δ^13^C is often smaller and provides limited separation. However, such comparisons are complicated by variation in isotope fractionation. For example, fluid-feeders (such as scale insects) and spiders may exhibit systematically lower δ^15^N values than other consumers [40–42].

No direct measures of fractionation are available for the organisms in our study, so we used a simple two-source isotope mixing model to verify that the large dietary contribution from the host plant suggested by our sequencing data would be consistent with plausible fractionation values for *A. usamba*. Assuming *A. usamba*’s diet comprises two sources: *C. mimosae* larvae and *V. drepanolobium* leaves, we can model *Anthene*’s stable isotope ratios δ^15^N_*Anth*_ and δ^13^C_*Anth*_ as a function of diet and fractionation:

$$\begin{array}{cc} \delta^{15}{\text{N}}_{Anth} & =p.\delta^{15}{\text{N}}_{\text{leaves}}+\left(1-p\right).\delta^{15}{\text{N}}_{\text{larvae}}+\Delta^{15}\text{N} \end{array}$$ (3)

$$\begin{array}{cc} \delta^{13}{\text{C}}_{Anth} & =q.\delta^{13}{\text{C}}_{\text{leaves}}+\left(1-q\right).\delta^{13}{\text{C}}_{\text{larvae}}+\Delta^{13}\text{C} \end{array}$$ (4)

where *p* and *q* are the fractions of assimilated nitrogen and carbon derived from leaves; δ^15^N_*i*_ and δ^13^C_*i*_ (where *i* = *Anth*, leaves, or larvae) are stable isotope ratios for the subscripted sources; and the fractionation values Δ^15^N and Δ^13^C are assumed to be constant across different sources in the diet. Substituting mean values for the δ^15^N_*i*_ and δ^13^C_*i*_ from our dataset into (3) and (4) yields linear relationships between *p* and Δ^15^N, and between *q* and Δ^13^C. We used these relationships to examine the Δ^15^N and Δ^13^C implied by *p* = *q* = 1.

## Results

### 16S amplicon sequencing

**Fig 1:**
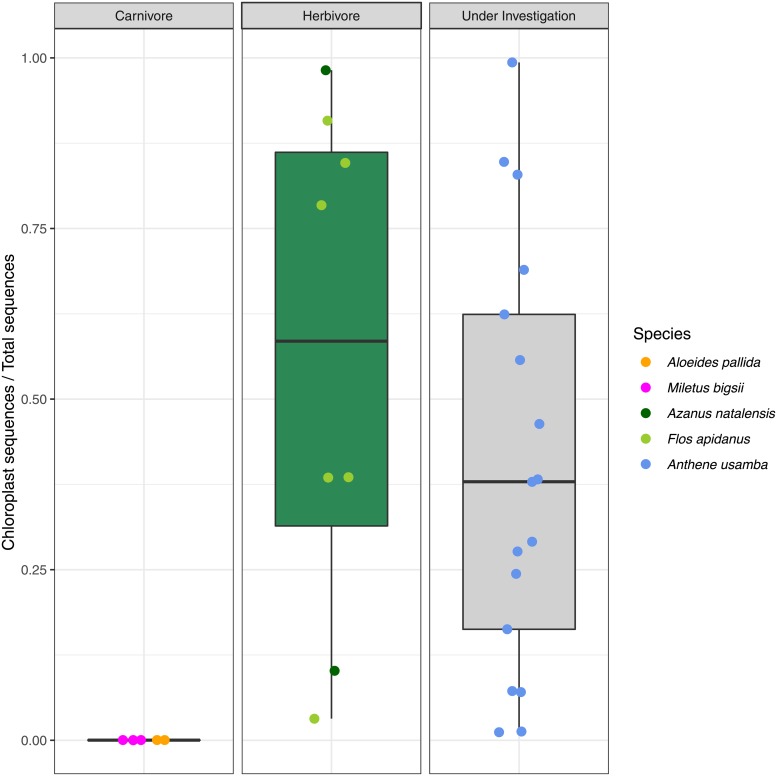
The sequence libraries of *Anthene usamba* caterpillars contained similar ratios of chloroplast to non-chloroplast sequences as those of phytophagous lycaenid species, whereas aphytophagous lycaenid species had no chloroplast sequences in their libraries.

The number of 16S sequences that passed quality filtering thresholds ranged from 15,526 to 46,273 sequences per sample, with an average of 29,078 sequences. Among the known phytophagous species—*Flos apidanus* and *Azanus natalensis*—chloroplast sequences comprised between 3% and 98% (median 58.4%, IQR 54.7%) of samples’ total libraries, whereas the libraries of entomophagous species—*Miletus biggsii* and *Aloeides pallida*—were confirmed to contain no chloroplast sequences (Fig 1). *Anthene usamba* sample libraries contained between 1% and 99% chloroplast sequences (median 37.8%, IQR 46.1%). A Kruskal-Wallis test demonstrated significant differences in chloroplast prevalence within the guts of caterpillars with different diets (*χ*^2^_(2)_ = 14.865, *p* < 0.01). Post hoc Wilcoxon rank sum tests showed that the difference in chloroplast prevalence between known carnivores and known herbivores was significant (*p* < 0.01), as was the difference between known carnivores and *A. usamba* (*p* < 0.01), whereas prevalence of chloroplasts in samples of *A. usamba* did not differ from those of known herbivores (*p* = 0.28).

### Plant barcoding

Plant chloroplast barcodes from the gut extracts of all *A. usamba* samples were identical and blasted with 100% identity to *Vachellia drepanolobium* Genbank accession KR738641 (S1 Fig) [43].

### Stable isotope analysis

**Fig 2:**
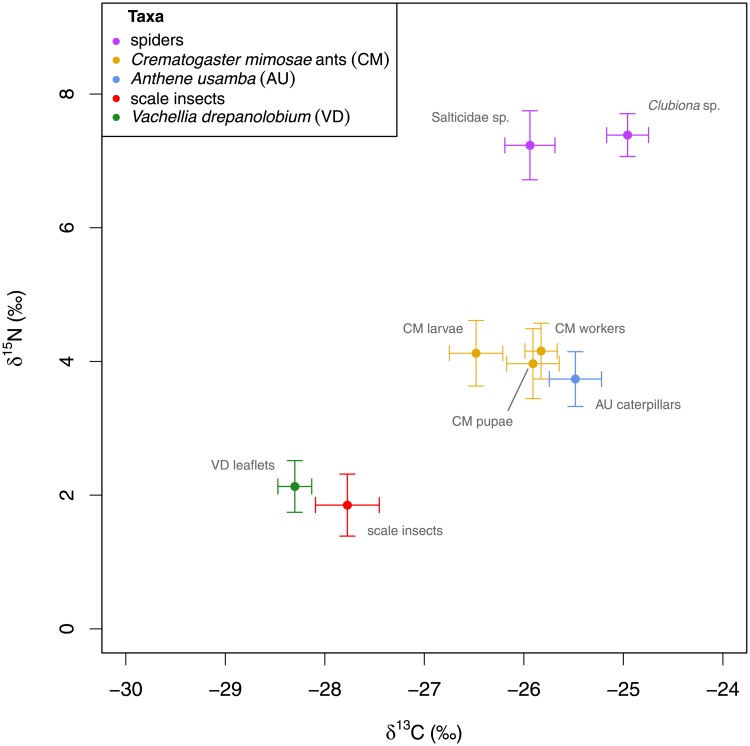
Stable isotope values are consistent with *C. mimosae* ants and *A. usamba* being functionally herbivorous on *V. drepanolobium*, while predatory Salticidae sp. and *Clubiona* sp. spiders are elevated by one trophic level. Error bars show standard errors.

Values of δ^15^N varied from around 2‰ for the samples of plant leaf tissue and scale insects, to about 7‰ for the two species of spider (Fig 2). The samples of *A. usamba* and *C. mimosae* returned δ^15^N values of approximately 4‰. *A. usamba* caterpillars were significantly higher in δ^15^N than *V. drepanolobium* leaves (*t*_44.8_ = 2.85, *p* < 0.01 for all samples, and *t*_15_ = 6.56, *p* < 0.01 for matched lycaenid/plant samples). *A. usamba* caterpillars and *C. mimosae* larvae showed marginally significant differences in δ^15^N (*t*_34.7_ = 0.60, *p* = 0.55 for all samples; but *t*_13_ = 2.07, *p* = 0.06 for matched lycaenid/ant samples). There was no significant difference in δ^15^N among *C. mimosae* larvae, pupae and workers (*F*_2,67_ = 0.04, *p* = 0.96).

Values of δ^13^C ranged from around -28‰ for the *V. drepanolobium* samples to about -25‰ for the *Clubiona* sp. spiders, consistent with the bulk of the carbon budget coming from the C_3_-photosynthesizing host plant rather than the C_4_-photosynthesizing grass understory, which we would expect to show values closer to -14‰ [44]. *A. usamba* caterpillars had significantly higher δ^13^C compared to *V. drepanolobium* leaves (*t*_30.4_ = 9.02, *p* < 0.01 for all leaves, and *t*_15_ = 12.44, *p* < 0.01 for matched lycaenid/plant samples). *A. usamba* caterpillars showed similar δ^13^C values to both *C. mimosae* workers and pupae (*A. usamba* vs. workers: *t*_28.3_ = 1.12, *p* = 0.27; *A. usamba* vs. pupae: *t*_34.8_ = 1.15, *p* = 0.26), but differed significantly from *C. mimosae* larvae (*A. usamba* vs. larvae: *t*_34.9_ = 2.66, *p* = 0.01). Consistent with this, *C. mimosae* workers, pupae and larvae showed marginally significant differences in δ^13^C (*F*_2,67_ = 2.44, *p* = 0.10), though it is possible that the inclusion of larval gut contents in those samples may contribute to this difference.

In comparison to other consumers of known trophic function, *A. usamba* caterpillars had significantly higher δ^15^N than scale insects (*t*_28.9_ = 3.05, *p* < 0.01), and significantly lower δ^15^N than *Clubiona* sp. or Salticidae sp. spiders (*t*_10.4_ = 7.02, *p* < 0.01 and *t*_21.3_ = 5.31, *p* < 0.01 respectively).

Our simple two-source mixing model suggests that high values of *p* and *q*, i.e. large fractions of assimilated nitrogen and carbon coming from leaves, would be consistent with plausible fractionation values. Rearranging (3) and (4) for the diet fractions *p* and *q*, and substituting mean empirical values for δ^15^N_•_ and δ^13^C_•_ gives

$$\begin{array}{cc} p & =(\frac{\delta^{15}{\text{N}}_{Anth}-\delta^{15}{\text{N}}_{\text{larvae}}-\Delta^{15}\text{N}}{\delta^{15}{\text{N}}_{\text{leaves}}-\delta^{15}{\text{N}}_{\text{larvae}}}) \end{array}$$ (5)

$$\begin{array}{cc} & =(\frac{3.74-4.12-\Delta^{15}\text{N}}{2.13-4.12}) \end{array}$$ (6)

$$\begin{array}{cc} & =0.19+0.50\Delta^{15}\text{N} \end{array}$$ (7)

$$\begin{array}{cc} q & =(\frac{\delta^{13}{\text{C}}_{Anth}-\delta^{13}{\text{C}}_{\text{larvae}}-\Delta^{13}\text{C}}{\delta^{13}{\text{C}}_{\text{leaves}}-\delta^{13}{\text{C}}_{\text{larvae}}}) \end{array}$$ (8)

$$\begin{array}{cc} & =(\frac{-25.5-\left(-26.5\right)-\Delta^{13}\text{C}}{-28.3-(-26.5)}) \end{array}$$ (9)

$$\begin{array}{cc} & =-0.55+0.55\Delta^{13}\text{C}. \end{array}$$ (10)

**Fig 3:**
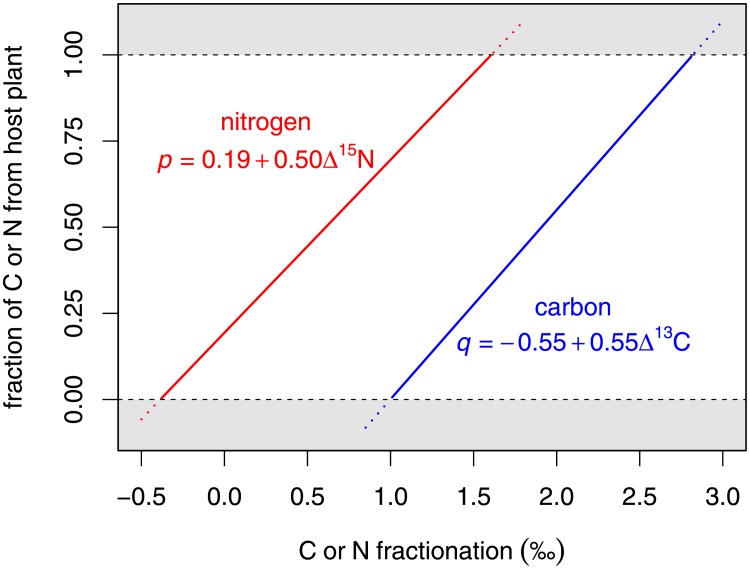
Estimated relationships between fraction of *A. usamba*’s nitrogen and carbon derived from *V. drepanolobium* (*p* and *q*, respectively), and *A. usamba*’s nitrogen and carbon isotopic fractionation (Δ^15^N and Δ^13^C, respectively) in a simple two-source mixing model.

If the host plant is the sole source of nitrogen and carbon, i.e. *p* = *q* = 1, these models suggest nitrogen fractionation of around 1.6‰ and carbon fractionation of around 2.8‰ (Fig 3).

## Discussion

Using an approach that incorporates evidence from DNA metabarcoding and stable isotope analysis, we assessed the diet and trophic position of *A. usamba* caterpillars, for which there are no direct observations of feeding behavior. We found that the larval guts of *A. usamba* and two known phytophagous lycaenid species contain chloroplast 16S sequences, and chloroplast barcoding recovered 100% sequence similarity between *V. drepanolobium* chloroplasts and the chloroplasts found in *A. usamba* guts. Stable isotope data, furthermore, are compatible with herbivory by *A. usamba* caterpillars.

Based on 16S sequence data, chloroplast prevalence is highly variable across caterpillars of confirmed phytophagous species. Whereas the sequence libraries of some phytophagous caterpillars are comprised of as much as 98% chloroplast sequences, others contain less than 2%. These values do not represent the absolute copy number of chloroplast 16S present in the larval guts, but rather the relative abundance of chloroplast versus non-chloroplast (i.e. bacterial) 16S sequences. However, no chloroplast 16S sequences were recovered from the guts of aphytophagous caterpillars.

Our nitrogen stable isotope results corroborate the metabarcode data, with *A. usamba* caterpillars showing δ^15^N values in the range that we would expect for herbivores on *V. drepanolobium*. *A. usamba*’s δ^15^N is similar to that for *C. mimosae*, which likely feeds on exudates from scale insects [45, 46]. Furthermore, *A. usamba*’s δ^15^N is lower than for two species of spider that we chose as exemplar minor predators in our system. Although fractionation rates may vary between spiders and insects on account of their different modes of nitrogen excretion [41, 42], potentially complicating this comparison, evidence suggests that fractionation in spiders tends to be at the lower end of the range seen in insects, which would strengthen the interpretation of our data as evidence for herbivory by *A. usamba*. *A. usamba*’s δ^15^N is also elevated compared to scale insects, which feed on *V. drepanolobium* fluids, but this relationship is not inconsistent with herbivory by *A. usamba*, as evidence suggests that fluid feeders tend to have low fractionation rates compared to other species [40]. While fractionation is unknown for *A. usamba*, our simple mixing model in (3) implies a δ^15^N of about 1.6 to be consistent with *A. usamba* obtaining the bulk of its nitrogen from the host plant. This value is well within the range of values known from the literature [40], including Lepidoptera: Tibbets *et al*., for example, report fractionation of 1.3 ± 0.4‰ in *Bombyx mori*, 1.4 ± 0.1‰ in *Manduca sexta* and 1.6 ± 0.5‰ in *Vanessa cardui* caterpillars [47].

The carbon stable isotope data are consistent with *A. usamba* obtaining the bulk of its carbon directly or indirectly from its C_3_-photosynthesizing host plant, rather than from grasses, which we would expect to substantially raise *A. usamba*’s δ^13^C. The value of δ^13^C given by our mixing model in (4) is consistent with *A. usamba* relying mainly on its host plant for carbon is within the range that we might expect from the literature, albeit towards the upper end of that range [40]. *A. usamba* and *C. mimosae* showed similarly elevated δ^13^C values compared to host plants or scale insects (Fig 2), which might reflect similar fractionation, a small contribution to the carbon budget of each species from C_4_ grasses [35] (though it is unclear what each of the species would be consuming in order for this contribution to occur), or tissue-specific variation in isotopic ratios [48].

We believe that the discrepancy between the results reported here and the previous estimation of *A. usamba*’s trophic position [9] can be explained in part by the use of different developmental stages for insect stable isotope analysis: Martins *et al*. (2013) conducted their isotopic analysis using wing clippings from adult *A. usamba* butterflies, while the present study analyzed the cuticles of *A. usamba* caterpillars. Working with adult butterflies provides many advantages, including ease of sampling and identifying adult butterflies in the field. Identifying lycaenid caterpillars to species based solely on morphology can be difficult or impossible, and the collection of *A. usamba* caterpillars requires laborious and destructive surveys of *V. drepanolobium* domatia. However, when using stable isotope analysis to estimate insect diets, selection of the appropriate developmental stage is crucial to avoid false conclusions. In the case of holometabolous insects such as butterflies, adult tissues are often enriched in ^15^N relative to larval tissues due to isotopic fractionation during metamorphosis [47, 49, 50]. Without knowledge of the size of this fractionation in *A. usamba*, the observed enrichment in ^15^N in *A. usamba* adults was interpreted as a shift in trophic position, when it is more likely to have been an artifact of ontogenetic changes.

Our results provide unambiguous evidence that *A. usamba* larvae consume *V. drepanolobium*, but the actual feeding behavior of the larvae remains unknown. In our surveys, we found *A. usamba* larvae in empty domatia as well as in domatia containing ant brood. In rare cases, we observed caterpillars in domatia containing fresh *V. drepanolobium* leaflets that were presumably brought in by ants. In no cases did we observe caterpillar frass inside the domatia, suggesting that caterpillars leave the domatia to defecate, and possibly to feed. Given that we have never observed caterpillars feeding, we suspect that they forage nocturnally, as has been observed in other ant-tended lycaenids (e.g., *Ogyris* [51]).

Is it possible that caterpillars might feed on ant brood or trophallaxis in addition to plant-feeding? We cannot rule this out from the sequencing or stable isotope data (especially given the uncertainty in Δ^15^N and Δ^13^C), but at the same time our results provide little evidence that it occurs. Nonetheless, with a few well-known exceptions, omnivory—consuming a diet that includes both plant and animal sources—is not common among lycaenids, although many phytophagous lycaenids are reported to cannibalize each other in their early instars, and collectors tend to be careful not to rear conspecifics together [7]. Species of two lycaenid genera, *Phengaris* (sometimes called *Maculinea*) in the Palearctic and *Lepidochrysops* in Africa, engage in phyto-predacious lifestyles in which caterpillars feed on plants for their first three instars, but complete their larval development feeding on ant brood or receiving food by trophallaxis. Whereas conspecific cannibalism is likely among *A. usamba* caterpillars, we think it unlikely that *A. usamba* caterpillars are phyto-predacious: early instar caterpillars have never been observed feeding on plants, and the present study sampled caterpillars that were in their 3rd instar or older, which would presumably comprise the aphytophagous stage of a phyto-predacious species’ development.

Further DNA barcoding surveys of the larval gut extracts using ant-specific primers could be carried out to assess the possibility of limited omnivory on ants as well as plants. However, this method would only test for presence of ant DNA in the larval guts, and would not be able to assess whether caterpillars feed on ant regurgitations. Ant regurgitations of extrafloral nectar would not explain the presence of plant DNA in the larval gut, but would likely have similar effects on *A. usamba*’s stable isotope profile as directly consuming plant tissue; careful experiments with isotopically labelled artificial nectar are likely to be a more promising way to identify any dietary contribution from trophallaxis. Finally, it is possible that caterpillars of *A. usamba* are facultative in their diet choice depending upon seasonal availability. The adults analyzed in our earlier study [9] were collected in late January, 2011, whereas the caterpillars analyzed for this study were collected in June, 2014. Caterpillars might widen their feeding preferences at different times of year and season, although this seems unlikely to be the only explanation for the large differences between the two studies.

This study highlights the power of using multiple sources of evidence when inferring the diets of animals with cryptic feeding habits, and corroborates the view that applying multiple methodologies yields more comprehensive assessments of animal diets (e.g., [52]). Both stable isotope analysis and DNA-based techniques have emerged as reliable methods for reconstructing the diets of species for which direct observations are lacking, but there are drawbacks to both approaches [5]. Reconstructing diets with stable isotope analysis requires prior knowledge of the isotopic signatures of potential food sources and an understanding of isotopic fractionation in the organism, tissue, and developmental stage of interest. Stable isotope methods cannot distinguish between food sources with similar isotopic profiles (e.g. plant material and ant regurgitations), and are less useful where the number of potential food sources exceeds the number of isotopes available for analysis [53]. DNA-based methods can suffer from PCR and database biases and difficulties in quantifying individual contributions to complex DNA mixtures, and can be misleading if there is differential degradation of various food sources within the gut [54–56]. Furthermore, analysis of DNA in an animal’s gut represents a single time point and cannot elucidate long-term dietary trends [57], nor can it detect food sources that do not leave a DNA signature, such as plant nectar, highly degraded detritus, acellular substrates like animal horn, or non-organic nutrient sources.

Given these drawbacks, stable isotope analysis and DNA metabarcoding are particularly complementary [2, 57, 58], and we suggest applying both techniques concurrently to avoid erroneous conclusions about food webs and ecosystem processes. In the case of lycaenids, for which diet and ant associations are crucial considerations in developing successful conservation plans for threatened species [59, 60], this dual approach can generate reliable natural history data with minimal cost and effort.

## Supporting information

S1 Table Samples included in each analysis. (PDF)

S1 File Butterfly COI barcoding. (PDF)

S1 Fig Representative sequence alignment showing 100% sequence similarity between plant trnL sequences generated from *Anthene usamba* gut contents and Genbank *Vachellia drepanolobium* accession KR738641. (EPS)

S2 Fig Stable isotope ratios δ^15^N and δ^13^C from Martins *et al*. (2013) overlaid on data from the current study. CMW: *C. mimosae* workers; CMP: *C. mimosae* pupae; CML: *C. mimosae* larvae.
(EPS)
