# Supplementary material for: Combining stable isotope analysis with DNA metabarcoding improves inferences of trophic ecology
Source: PLoS One. 2019 Jul 22;14(7):e0219070. doi: 10.1371/journal.pone.0219070 (PMC6645532; doi:10.1371/journal.pone.0219070)
Supplement: S1 Table — (PDF) [file pone.0219070.s001.pdf]

Sample information

Table S1. Samples included in each analysis.

Sample ID	Description	16S	SIA	COI	trnL
MRLW_K5_2014	*Anthene usamba* - Gut	-	X	X	-
MRLW_K11_2014	*Anthene usamba* - Gut	X	X	X	X
MRLW_K18_2014	*Anthene usamba* - Gut	-	X	X	-
MRLW_K22_2014	*Anthene usamba* - Gut	X	X	X	-
MRLW_K39_2014	*Anthene usamba* - Gut	X	X	X	X
MRLW_K56_2014	*Anthene usamba* - Gut	X	X	X	-
MRLW_K60_2014	*Anthene usamba* - Gut	X	X	X	X
MRLW_K64_2014	*Anthene usamba* - Gut	X	X	X	X
MRLW_K65_2014	*Anthene usamba* - Gut	X	X	X	X
MRLW_K66_2014	*Anthene usamba* - Gut	X	X	X	-
MRLW_K75_2014	*Anthene usamba* - Gut	X	X	X	X
MRLW_K76_2014	*Anthene usamba* - Gut	X	X	X	X
MRLW_K77_2014	*Anthene usamba* - Gut	X	X	X	-
MRLW_K78_2014	*Anthene usamba* - Gut	-	X	X	-
MRLW_K90_2014	*Anthene usamba* - Gut	X	X	X	X
MRLW_K91_2014	*Anthene usamba* - Gut	X	X	X	X
MRLW_K92_2014	*Anthene usamba* - Gut	X	X	X	X
MRLW_K200_2014	*Anthene usamba* - Whole	X	-	X	X
MRLW_K201_2014	*Anthene usamba* - Whole	X	-	-	-
MRLW_K213_2014	*Anthene usamba* - Whole	X	-	X	X
MRLW_S01_2014	*Flos apadanus* - Gut	X	-	-	-
MRLW_S03_2014	*Flos apadanus* - Gut	X	-	-	-
MRLW_S04_2014	*Flos apadanus* - Gut	X	-	-	-
MRLW_S05_2014	*Flos apadanus* - Gut	X	-	-	-
MRLW_S06_2014	*Flos apadanus* - Gut	X	-	-	-
MRLW_S22_2014	*Flos apadanus* - Gut	X	-	-	-
MRLW_S25_2014	*Miletus bigsii* - Whole	X	-	-	-
MRLW_S26_2014	*Miletus bigsii* - Gut	X	-	-	-
MRLW_S27_2014	*Miletus bigsii* - Gut	X	-	-	-
MRLW_S31_2014	*Miletus bigsii* - Gut	X	-	-	-
MRLW_SA01_2014	*Aloeides pallida* - Gut	X	-	-	-
MRLW_SA02_2014	*Aloeides pallida* - Gut	X	-	-	-
MRLW_SA113_2014	*Azanus natalensis* - Whole	X	-	X	-
MRLW_SA114_2014	*Azanus natalensis* - Whole	X	-	X	-
