# Supplementary material for: Combining stable isotope analysis with DNA metabarcoding improves inferences of trophic ecology
Source: PLoS One. 2019 Jul 22;14(7):e0219070. doi: 10.1371/journal.pone.0219070 (PMC6645532; doi:10.1371/journal.pone.0219070)
Supplement: S1 File — (PDF) [file pone.0219070.s002.pdf]

Butterfly COI barcoding

Since lepidopteran caterpillars are often challenging to identify to species, we used COI barcodes to confirm the identities of our larval *Anthene* specimens against identified adult *Anthene* spp. voucher samples deposited at the Museum of Comparative Zoology (MCZ), Harvard University and against published sequences from GenBank [1] and the Barcode of Life Database (BOLD [2]) (Table 1).

We extracted DNA from larval *Anthene* samples used for gut contents metabarcoding as described in the manuscript's Methods. We performed additional extractions from *Anthene* samples used in the stable isotope analysis with a standard phenol-chloroform protocol, and from the MCZ samples using the Qiagen Blood and Tissue kit (Qiagen Inc., Germantown, Maryland, USA) according to manufacturer's instructions. We amplified the COI barcode region using primers LCO1490 (5'-GGTCAACAAATCATAAAGATATTGG-3') and HCO2198 (5'-TAAACTTCAGGGTGACCAAAAAATCA-3') [3].

We performed PCRs using 12.5μL Omega 2X MasterMix (Bio-Tek, Norcross, Georgia, USA), 0.5mM $MgCl_2$, 0.5μM each primer, 1μL template and water to 25μL. PCR conditions followed a touchdown profile: 3 min at 94°C, followed by 20 cycles of 50 sec at 94°C / 40 sec at 49°C decreasing by 0.5°C per cycle / 80 sec at 72°C, followed by a further 20 cycles of 50 sec at 94°C / 40 sec at 48°C / 80 sec at 72°C, and a final 5 min at 72°C. PCR products were either purified using an ExoAP digest and ethanol precipitation before sequencing on an ABI 3130xl capillary sequencer (MCZ samples and additional stable isotope *Anthene* samples), or were sent to ETON Bioscience (Boston, Massachusetts, USA) for purification and sequencing (metabarcoding *Anthene* samples). Edited sequences were deposited in GenBank with accession numbers MK862031 – MK862083.

All *Anthene* sequences available on BOLD and GenBank were included in the analysis. We selected non-*Anthene* reference sequences for inclusion using sequence searches in the Barcode of Life animal identification tool and GenBank BLAST of larval and the two identified *Anthene hodsoni* (=*A. usamba*) sequences. We aligned sequences globally using the Geneious alignment algorithm as implemented in Geneious 9.1.3 (https://www.geneious.com) with free end gaps and a 65% similarity cost matrix. We generated a maximum likelihood tree using PhyML [4] as implemented in Geneious 9.1.3 using the GTR+G model with best of nearest-neighbor interchange and subtree pruning-regrafting tree topology searches.

The putative *A. usamba* larval samples from this study were placed close to MCZ specimen DJM_10_A449 (Fig 1). These specimens together form a monophyletic group with close genetic relationships among the specimens, confirming that the caterpillars investigated in this manuscript belong to a single species. The wings of MCZ specimen DJM_10_A449 are consistent with its previous identification as *A. hodsoni* (=*A. usamba*) which, alongside the phylogenetic analysis, identifies our larval samples as *A. usamba.*

There are two MCZ wing vouchers identified as *A. hodsoni* (=*A. usamba*). The wing vouchers for DJM_06_K497 are distinctly different from those for DJM_10_A449, and the molecular data indicate that these specimens are relatively divergent. The BOLD animal identification tool identifies DJM_06_K497 as *Deudorix ecuadata* (BOLD specimen PMANL3874-15) with 99.69% similarity. Phylogenetic analysis also places it outside of the *Anthene* clade and, therefore, we consider this specimen to be misidentified. Two additional *Anthene* (*A. butleri*) specimens also fell into the outgroup clade, highlighting the difficulty of morphological species identifications in these genera. Indeed, even within the *Anthene* clade, several taxa appear non-monophyletic in our phylogenetic tree, and may represent taxa in need of revision or additional genetic resolution. *Anthene* is a highly speciose and poorly-resolved genus, and further work to resolve the phylogeny would be well worthwhile. Nonetheless, the resolution of taxonomy and

relationships among these taxa appears unlikely to affect our conclusions regarding the monophyly of the *A. usamba* larval samples and MCZ specimen DJM_10_A449.

Two caterpillars were identified as *Azanus natalensis* (MRLW_SA113_2014 and MRLW_SA114_2014) using the BOLD species identification tool (99.33% similarity) and phylogenetic analysis.

Table 1. Sequences and specimens used for COI barcoding.

Sample ID	Source	Species
MRLW_K5_2014, MRLW_K11_2014, MRLW_K18_2014, MRLW_K22_2014, MRLW_K39_2014, MRLW_K56_2014, MRLW_K60_2014, MRLW_K64_2014, MRLW_K65_2014, MRLW_K66_2014, MRLW_K75_2014, MRLW_K76_2014, MRLW_K77_2014, MRLW_K78_2014, MRLW_K90_2014, MRLW_K91_2014, MRLW_K92_2014, MRLW_K200_2014, MRLW_K213_2014	this study	*Anthene usamba*
DJM_07_A057, DJM_07_A060	MCZ	*Anthene amarah*
LSER018-06, LSER035-06, LSER009-06	BOLD	*Anthene amarah*
LSER012-06, LSER036-06	BOLD	*Anthene butleri*
DJM_10_A232	MCZ	*Anthene contrastata*
LSER233-06	BOLD	*Anthene contrastata*
GWOTD818-12, LSAFR1185-12, LSAFR1186-12	BOLD	*Anthene definita definita*
DJM_06_J967, DJM_07_A085, DJM_10_A326	MCZ	*Anthene definita*
GBGLL606-14, GBMIN81721-17, KHCBT1124-16, GBGL1353-06	BOLD	*Anthene emolus*
KX151716.1, KT286518.1	GenBank	*Anthene emolus*
DJM_06_K497, DJM_10_A449	MCZ	*Anthene hodsoni* (=*A. usamba*)
DJM_06_I957, DJM_10_A335, DJM_07_A026	MCZ	*Anthene indefinita*
DJM_10_A258	MCZ	*Anthene larydas*
DJM_10_A450, DJM_10_A260, DJM_10_A423, DJM_10_A428, DJM_07_A008	MCZ	*Anthene ligures*
DJM_07_A056	MCZ	*Anthene lunulata*
LOQTI473-11, ANICT302-11	BOLD	*Anthene lycaenoides*
DJM_12_Q106	MCZ	*Anthene otacilia*
DJM_10_A305	MCZ	*Anthene rubricinctus*
DJM_10_A464, DJM_10_A303, DJM_07_A077, DJM_10_A304, DJM_10_A415, DJM_07_A078, DJM_10_A427, DJM_07_A179, DJM_10_A461, DJM_10_A460, DJM_07_A176	MCZ	*Anthene schoutedeni*
ANICT297-11, ANICT298-11, ANICT299-11	BOLD	*Anthene seltuttus*
GBMIN32677-13	BOLD	*Anthene staudingeri*
BCIBT815-13, GWOTT922-18	BOLD	*Arawacus lincoides*
MRLW_SA113_2014, MRLW_SA114_2014	this study	*Azanus natalensis*
LSER051-06, LSER116-06	BOLD	*Azanus natalensis*
KP871115, KP053283	GenBank	*Azanus jesous*
GQ129014	GenBank	*Azanus mirza*
KY839025	GenBank	*Azanus* sp.
KY839025	GenBank	*Azanus* sp.
EZBNB046-08, EZBNB047-08	BOLD	*Callophrys polios*
PMANL3874-15	BOLD	*Deudorix ecuadata*
GWOSQ302-11, GWORA2831-15	BOLD	*Deudorix livia*
MF546256.1	GenBank	*Ministrymon una*
KU380740.1	GenBank	*Panthiades boreas*
KU380741.1	GenBank	*Panthiades ochus*
GU162907.1	GenBank	*Symbiopsis tanais*

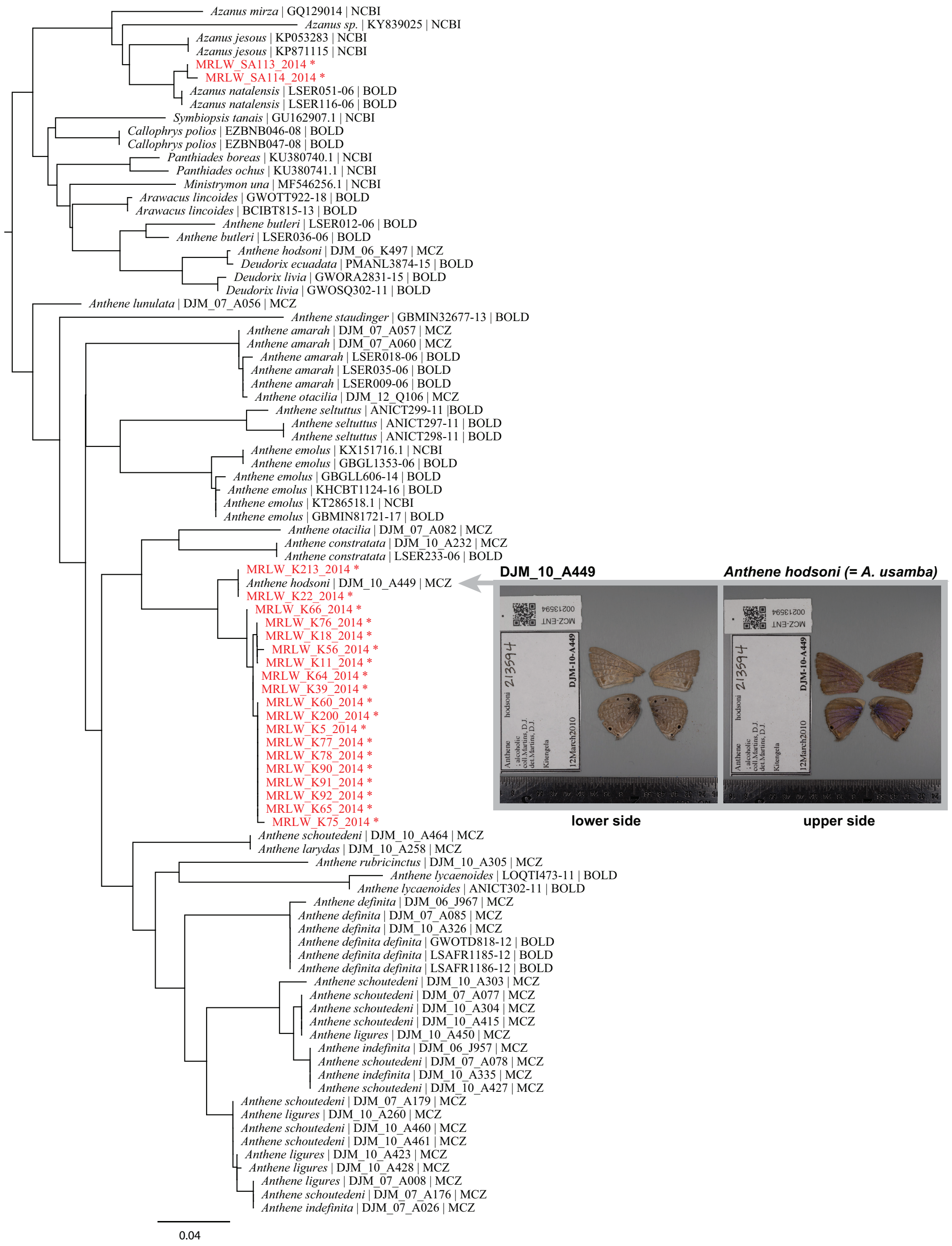

Fig 1. Phylogenetic analysis of larval samples analyzed in this paper (denoted with * and colored red), alongside reference sequences obtained from GenBank, the Barcode of Life Database (BOLD), and Harvard's Museum of Comparative Zoology (MCZ). Phylogeny was estimated based on mitochondrial cytochrome c oxidase subunit I (COI) gene sequences using a Geneious global alignment and maximum likelihood tree building. Tip labels for reference sequences show species names and sample identifiers as provided by the reference collections. This analysis identifies our larval samples as *Azanus natalensis* (MRLW_SA113_2014 and MRLW_SA114_2014) and *Anthene usamba* (all other samples). These *A. usamba* samples form a monophyletic group with MCZ specimen DJM_10_A449, whose wing vouchers, pictured, are consistent with identification by the collector as *Anthene hodsoni* (= *A. usamba*).

References

1. Benson DA, Karsch-Mizrachi I, Lipman DJ, Ostell J, Wheeler DL. GenBank. Nucleic Acids Research. 2005;33:D34–D38.

2. Ratnasingham S, Hebert PDN. BOLD: The Barcode of Life Data System (http://www.barcodinglife.org) Molecular Ecology Notes. 2007;33(7):355–364.

3. Folmer O, Black M, Hoeh W, Lutz R, Vrijenhoek R. DNA primers for amplification of mitochondrial cytochrome *c* oxidase subunit I from diverse metazoan invertebrates Molecular Marine Biology and Biotechnology. 1994;3(5):294–299.

4. Guindon S, Gascuel O. A simple, fast, and accurate algorithm to estimate large phylogenies by maximum likelihood Systematic Biology. 2003;52(5):696–704.
